# Gastritis cystica profunda with early gastric cancer persisting for 9 years in the stage of high-grade intraepithelial neoplasia

**DOI:** 10.1186/s12876-026-05202-9

**Published:** 2026-08-10

**Authors:** Jianling Song, Ruoxuan Hou, Lan Yang, Haijun Zhang, Yi Zheng

**Affiliations:** 1 Department of Gastroenterology, The First Affiliated Hospital of Shihezi University, 107 Beier Road, Shiheizi, Xinjiang 832008 China; 2 Shihezi University, 221 Beisi Road, Shihezi, Xinjiang 832003 China; 3 Department of Pathology, First Affiliated Hospital, Shihezi University, 107 Beier Road, Shihezi, Xinjiang 832008 China; 4 Department of Gastroenterology, Shihezi People’s Hospital, 411 Naner Xiyi Road, Shihezi, Xinjiang 832000 China

**Keywords:** Gastritis cystica profunda, Early gastric cancer, Endoscopic submucosal dissection, High-grade intraepithelial neoplasia

## Abstract

Gastritis cystica profunda (GCP) is a benign lesion characterized by the submucosal proliferation of cystic gastric glands. It most frequently occurs in patients with a history of gastric surgery but is infrequently found in those with an unoperated stomach. While generally benign, GCP has been associated with gastric adenocarcinoma in some cases. We report a case of early gastric cancer (EGC) arising from GCP in a 69-year-old man with no history of gastric surgery. The lesion, which had remained in the stage of high-grade intraepithelial neoplasia for nine years, was successfully treated with endoscopic submucosal dissection (ESD). This case highlights the diagnostic challenges posed by GCP-associated lesions and suggests that such lesions may exhibit an indolent clinical course. In 2016, a gastroscopic examination revealed a 0-IIa + IIc lesion of approximately 40 mm in diameter on the greater curvature of the gastric antrum, close to the posterior wall. A biopsy confirmed high-grade intraepithelial neoplasia. The lesion was deemed indicated for ESD. However, during the procedure, a poor lifting sign following submucosal injection led to the suspicion of submucosal infiltration, and the treatment was subsequently aborted. The patient declined further treatment and opted for clinical follow-up. In 2025, a follow-up gastroscopy showed no significant change in the lesion compared to its 2016 appearance. A second ESD was successfully performed. Histopathological examination of the resected specimen confirmed the diagnosis of GCP with well-differentiated adenocarcinoma (confined to high-grade intraepithelial neoplasia). All lateral and deep margins were free of neoplasia, with no evidence of lymphatic, vascular, or perineural invasion. The surrounding gastric epithelium showed normal mucosa without dysplasia. This case highlights the diagnostic challenges posed by GCP-associated lesions and suggests that, in selected instances, such lesions may follow a relatively indolent clinical course.

## Introduction

Gastritis cystica profunda (GCP) is a benign lesion characterized by the misplacement of gastric mucosal glands into the submucosa and occasionally extending to the muscularis propria [1, 2]. Although this lesion most commonly develops in patients with a history of gastroenterostomy, it can also occur in unoperated stomachs where widespread chronic active or atrophic gastritis is considered a significant predisposing factor [2, 3].

Emerging evidence suggests that GCP may be associated with gastric carcinoma or adenoma exhibiting high-grade dysplasia [4]. Notably, recent investigations have revealed a negative correlation between GCP and lymph node metastasis (LNM) risk in early gastric cancer (EGC) [5]. However, the clinical significance and natural history of GCP-associated malignancies remain incompletely characterized. This protective association remains consistent across diverse tumor characteristics including location, size, ulceration status, differentiation grade, depth of invasion, and lymphovascular involvement. These findings collectively indicate that GCP-associated gastric malignancies tend to demonstrate an indolent biological behavior. While endoscopic ultrasonography may reveal submucosal thickening in GCP that can be misinterpreted as malignant infiltration, endoscopic submucosal dissection (ESD) remains the indicated therapeutic approach for this condition [6].

We herein present a case of early gastric cancer arising from GCP in a 69-year-old man without prior gastric surgery. The lesion, which maintained high-grade intraepithelial neoplasia for nine years, was successfully treated with ESD. Histopathological examination confirmed that the carcinoma cells were confined to the mucosal layer without submucosal invasion.

## Case report

**Fig. 1:**
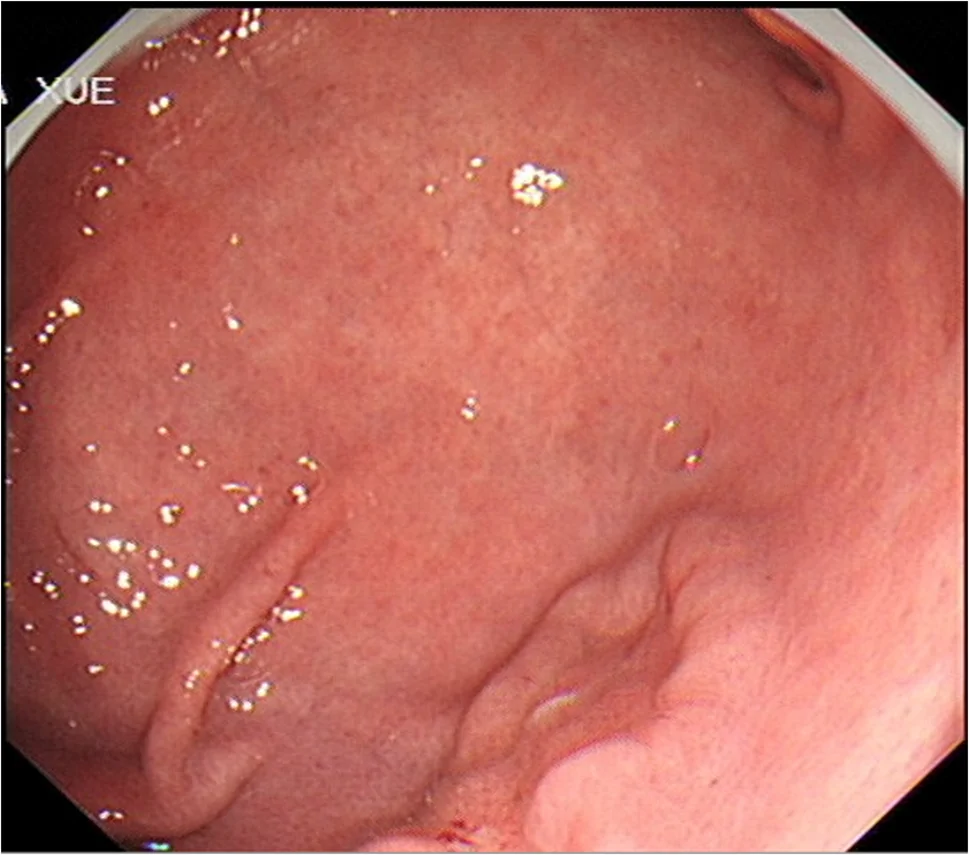
A type IIa + IIc lesion on the posterior wall of the gastroduodenal junction in 2016

**Fig. 2:**
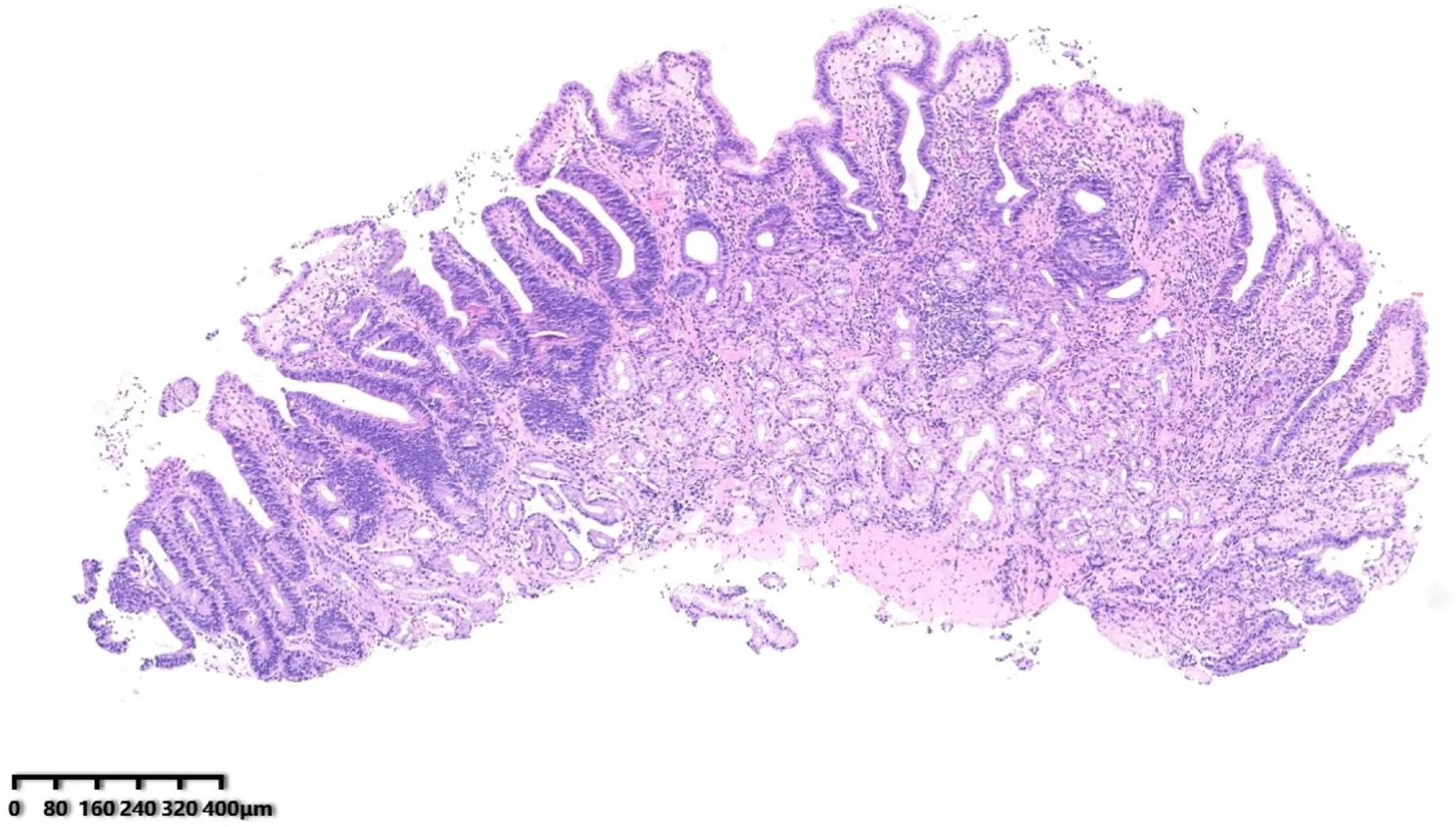
Biopsy confirmed high-grade intraepithelial neoplasia

**Fig. 3:**
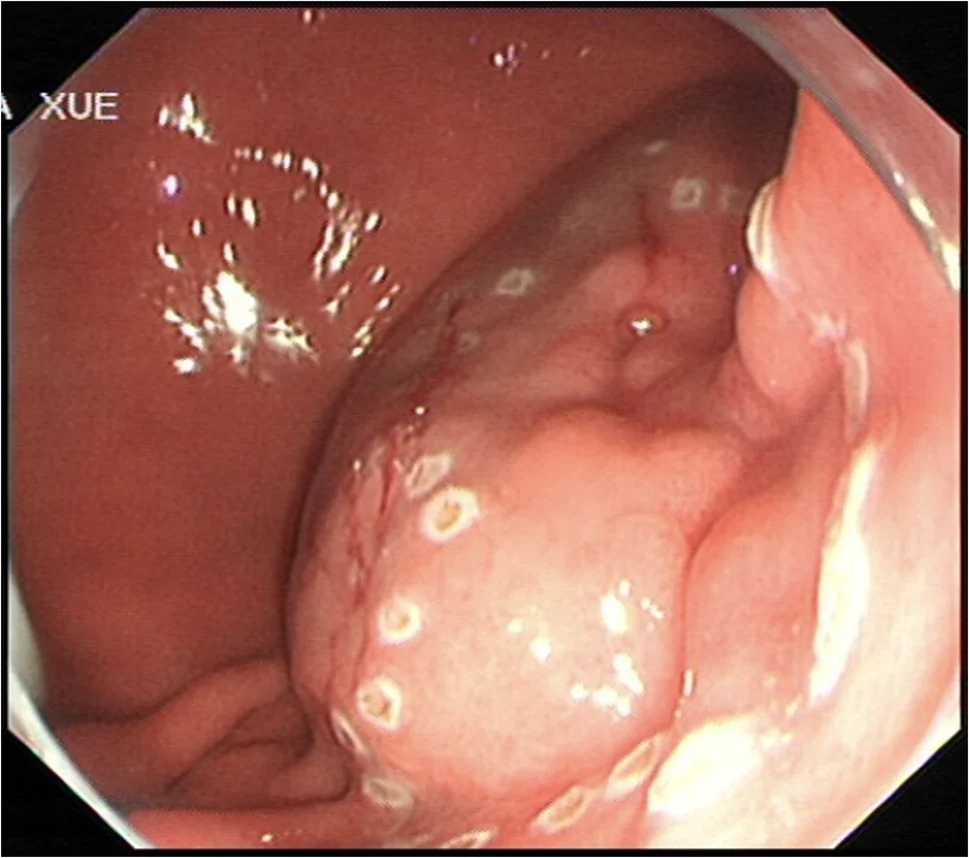
A poor lifting sign in the central portion of the lesion after submucosal injection

**Fig. 4:**
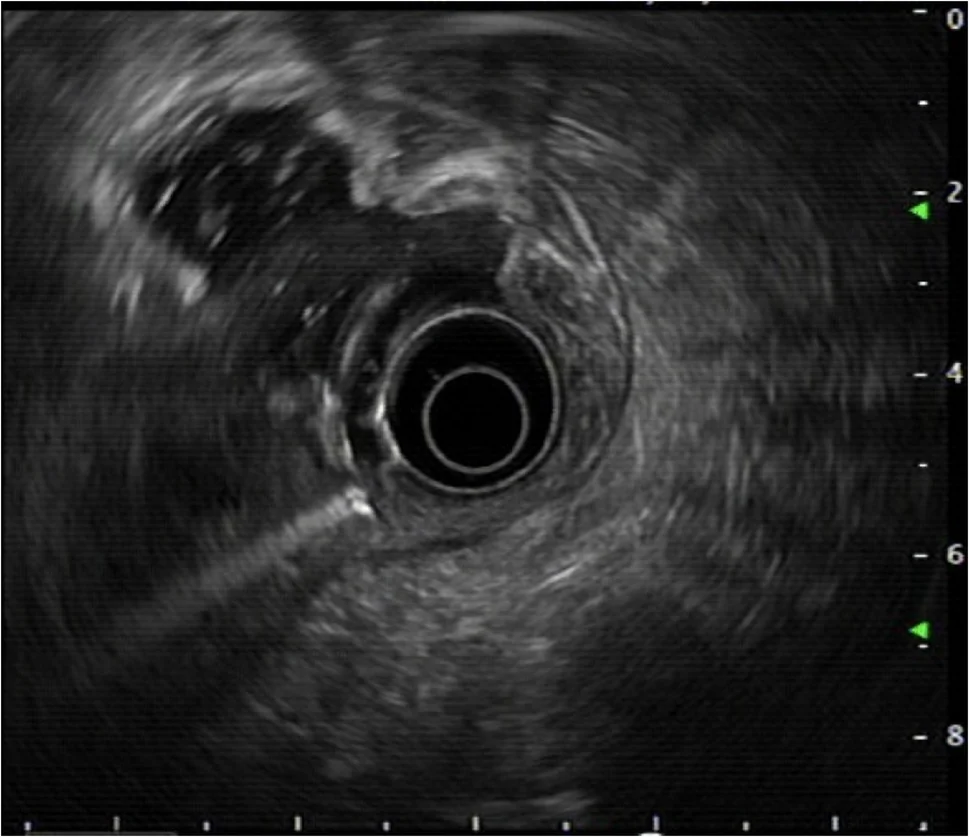
Endoscopic ultrasonography (6 MHz) showing thickening of the submucosal layer

**Fig. 5:**
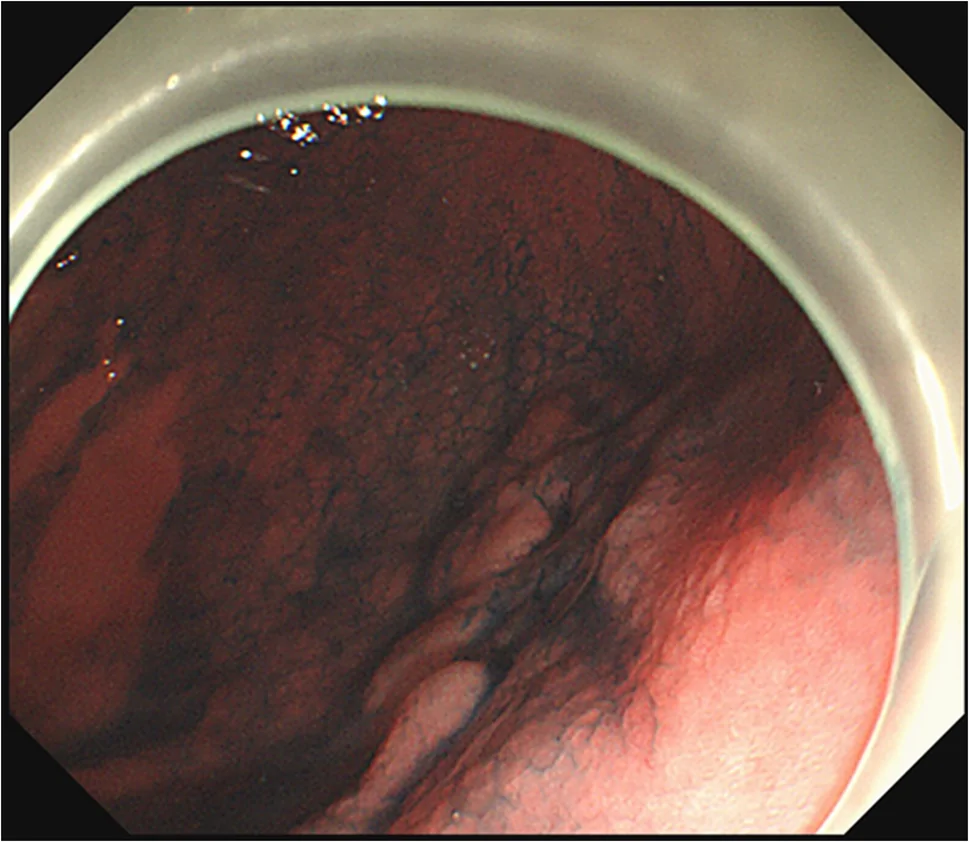
No significant change in the lesion compared to the 2016 findings

**Fig. 6:**
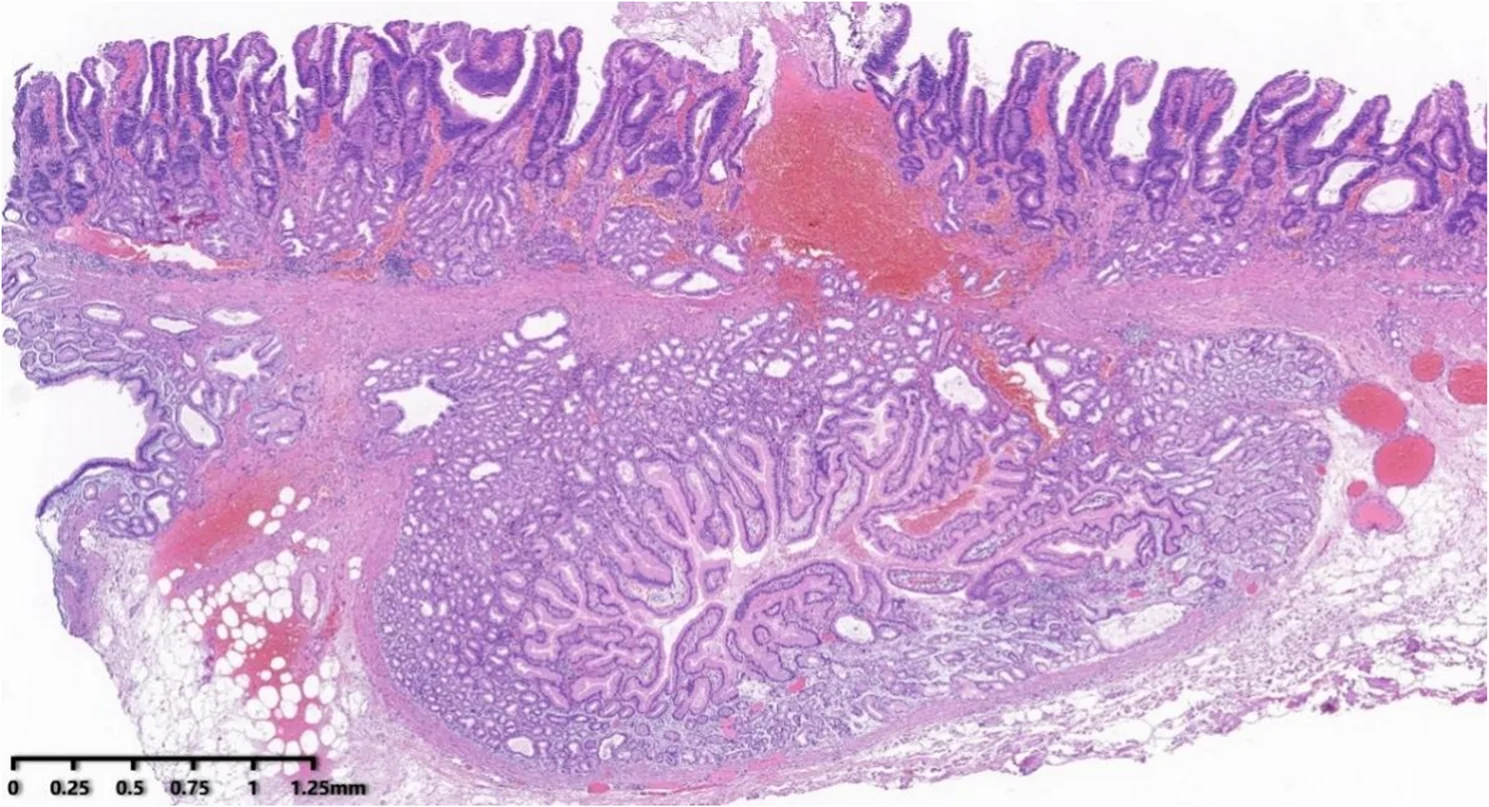
Dilated glands extending into the submucosa

**Fig. 7:**
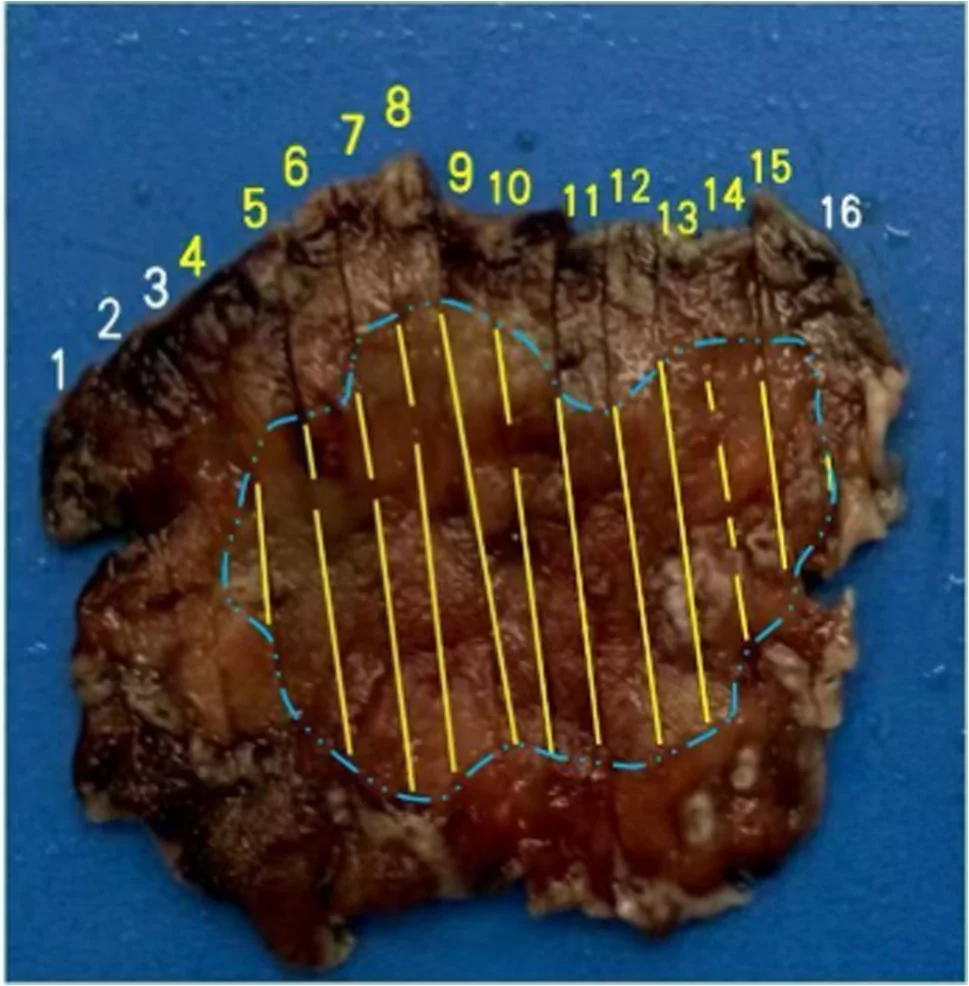
Gross photograph of the ESD‑resected specimen. Blue dotted line: boundary of high‑grade intraepithelial neoplasia (HGIN); yellow lines: serial sectioning lines (numbered 1–16) corresponding to the histological strips shown in Fig. 8

**Fig. 8:**
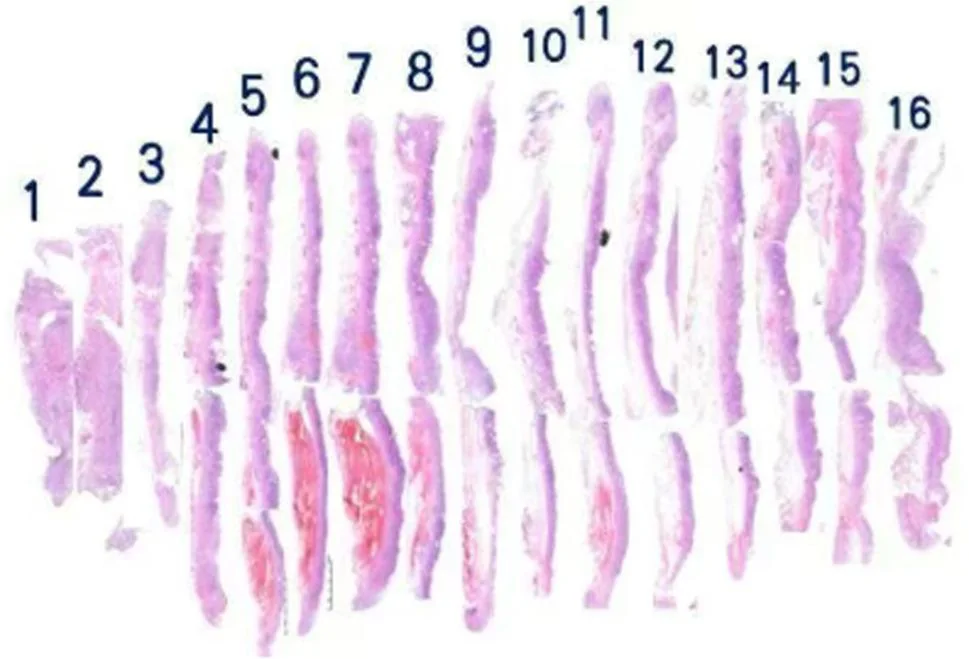
Serial histological strips (Sects.  1–16, H&E) of the resected specimen, corresponding to the sectioning lines in Fig. 7

**Fig. 9:**
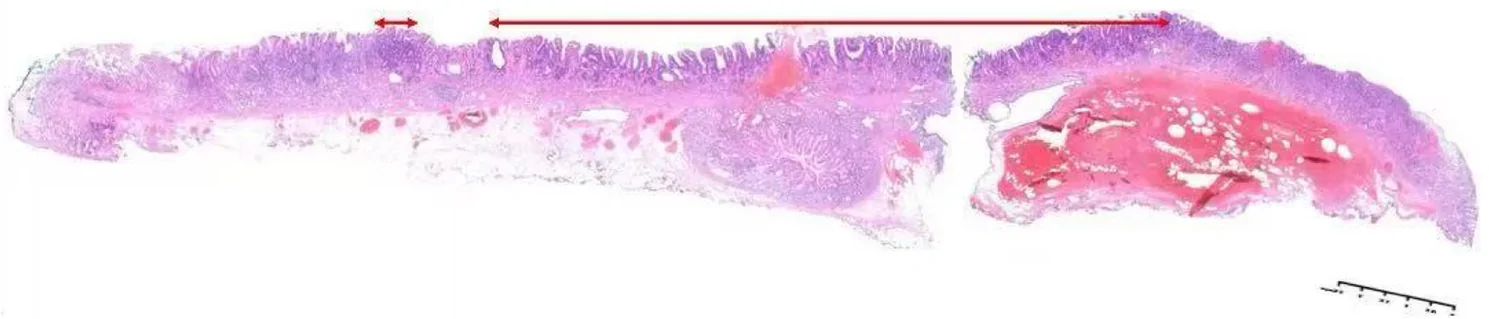
Representative panoramic histology (H&E) of the lesion. Short arrow: High-grade intraepithelial neoplasia(HGIN) without submucosal gastritis cystica profunda (GCP) glands; long arrow: (HGIN) overlying submucosal GCP glands

A 60-year-old male patient was found to have a gastric mucosal lesion during a routine upper gastrointestinal endoscopy in 2016. The patient was in good clinical health with no family history of gastrointestinal diseases or malignant tumors. He reported no abdominal discomfort or gastrointestinal symptoms. Physical examination revealed no abnormalities, and initial biochemical and hematological parameters were within normal limits, with a normal serum CEA level. The C14 urea breath test detected a positive result for Helicobacter pylori infection. The surrounding gastric mucosa showed endoscopic signs of chronic atrophic gastritis, including decreased mucosal folds and visible vascular patterns. Histological examination of biopsy samples from the antrum and corpus revealed mild to moderate chronic active inflammation with mononuclear cell infiltration, consistent with H. pylori‑associated gastritis. No intestinal metaplasia was identified in the surrounding mucosa. Endoscopic examination identified a type IIa + IIc lesion on the posterior wall of the gastroduodenal junction (Fig. 1). Magnifying endoscopy with narrow‑band imaging revealed a clear demarcation line corresponding to the depressed area, with irregular microvascular and microsurface patterns suggestive of neoplasia. In contrast, the elevated component of the lesion showed relatively preserved glandular and vascular architectures without obvious irregularity, although mild dilatation of intervening capillaries was noted. A biopsy confirmed high-grade intraepithelial neoplasia (Fig. 2). Based on these findings, the lesion was considered an absolute indication for ESD. After obtaining informed consent from the patient’s family, ESD was initiated. During the procedure, a poor lifting sign was observed following submucosal injection in the central portion of the lesion (Fig. 3), raising suspicion of submucosal invasion. Subsequent EUS was performed at 6 MHz (Fig. 4). EUS revealed asymmetric thickening of the submucosal layer with heterogeneous echogenicity. However, no definitive anechoic cystic structures characteristic of typical GCP were identified within the thickened submucosa, possibly due to the relatively small size of the cystic glands or limitations of the imaging resolution. Given the concern for invasive carcinoma, the ESD procedure was discontinued. As the patient remained asymptomatic, he opted for regular follow-up. In 2025, the patient presented again with complaints of upper abdominal fullness and discomfort. Repeat endoscopy showed no significant change in the lesion compared to the 2016 findings (Fig. 5), suggesting that the lesion might not be invasive. With the patient’s consent, a second attempt at ESD was made. Although a poor lifting sign was again noted after submucosal injection, the lesion was successfully resected en bloc. Histopathological examination of the resected specimen revealed high-grade intraepithelial neoplasia in the mucosal epithelium, with dilated glands extending into the submucosa (Fig. 6). The lateral and deep margins were free of tumor involvement, and there was no evidence of lymphatic, vascular, or perineural invasion. The histopathological findings were consistent with early gastric cancer originating from gastritis cystica profunda (GCP), and the lesion was considered curatively resected. The resected ESD specimen was serially sectioned for detailed histopathological mapping (Figs. 7, 8 and 9). The gross specimen showed a slightly elevated mucosal lesion (Fig. 7). Serial histological sections (Fig. 8) and representative panoramic histology (Fig. 9) confirmed that the HGIN was localized to the mucosal epithelium overlying the central and slightly eccentric portion of the lesion, while the cystically dilated glands of GCP extended into the submucosa beneath and immediately adjacent to the area of HGIN, with some cystic glands located beyond the lateral margins of the neoplastic epithelium. All resection margins were negative for neoplasia.

## Discussion and conclusions

To date, several studies have retrospectively analyzed the natural course of gastric cancer, indicating that early-stage gastric cancer follows a relatively prolonged natural history, while advanced disease demonstrates significantly accelerated progression. A 2000 Japanese study elucidated the natural history of early gastric cancer (EGC): among 56 cases followed for 6-137 months, 20 remained in the early stage, while 36 progressed to advanced disease. The proportion of cases remaining in the early stage declined continuously over time, with a median duration of approximately 44 months [7]. Another study reported that the doubling time shortened with tumor progression, and the transition from EGC to advanced gastric cancer took about 34 months [8].

Notably, the present case remained in the early stage far longer than this median duration. This observation raises the question of what factors might have contributed to such prolonged early stage persistence. Beyond favorable tumor differentiation, the coexistence of GCP in this case might have contributed to the prolonged course, although this remains speculative.

GCP was first described by Littler [1] in 1972 as “gastritis cystica polyposa,” referring to gastric mucosal glands herniating through the muscularis mucosae, forming cystic expansions in the submucosa, and occasionally extending into the muscularis propria. Later, Franzin et al. [9] analyzed 37 similar cases and noted morphological similarities to colitis cystica profunda—a condition featuring submucosal mucus-filled cysts in the colon—but without cystic fluid content, thus terming it “gastritis cystica profunda.” As various etiologies may lead to this condition, it is now defined as a disorder wherein gastric mucosal glands (foveolar or proper glands) are misplaced into the muscularis mucosae, submucosa, or even deeper layers (muscularis propria or serosa), due to multiple potential causes.

GCP is clinically rare and nonspecific, often with normal serological findings. Endoscopically, it presents as multiple polyps or elevated lesions. However, due to limited biopsy depth and sample size, conventional endoscopy rarely achieves a definitive diagnosis. With advances in endoscopic technology, endoscopic ultrasonography (EUS) can aid in diagnosis, though histopathology remains the gold standard. Typical EUS features of GCP include: lesion origin in the gastric mucosal layer, localized wall thickening, and irregular anechoic or hypoechoic areas extending into the submucosa and beyond—either unilocular or multilocular—with an intact mucosal surface. Pathologically, GCP is characterized by elongated gastric pits, hyperplastic and cystically dilated glands extending into the submucosa, usually with normal gland morphology [10]. Chronic inflammation and eosinophilic infiltration are common accompaniments. Immunohistochemically, the lesions typically show wild-type p53 expression and a Ki-67 proliferation index of < 1% [11].

Early gastric cancers may also display normal overlying mucosa. Well-differentiated adenocarcinoma, in particular, shows minimal cytological and structural deviation from normal, making it difficult to distinguish from GCP when there is submucosal invasion [5]. When GCP is accompanied by neoplasia, the cystically dilated glands cause mucosal elevation, which can significantly interfere with accurate assessment of tumor nature and depth.

This clinical course underscores a diagnostic pitfall: the elevated mucosa and submucosal thickening led to endoscopic overestimation of invasion depth, resulting in an erroneous decision to abort ESD and refer the patient for surgical consultation. Moreover, the patient’s nine‑year interval without interval imaging reflects the limited clinical awareness of GCP’s potential for indolent behavior. Nevertheless, the eventual successful ESD confirmed that this GCP‑associated lesion remained confined to the mucosal layer. This experience aligns with previous observations that gastric cancers with coexisting GCP often present at earlier stages and carry a lower metastatic potential [12], reinforcing that the presence of GCP should not automatically prompt escalation to surgery without careful endoscopic evaluation.

The poor lifting sign observed during both ESD attempts in this case is noteworthy. While submucosal fibrosis due to prior biopsy might contribute to this phenomenon, the persistence of poor lifting over nine years without significant interval change argues against biopsy‑induced fibrosis as the sole explanation. Instead, the submucosal cystic glandular proliferation characteristic of GCP likely resulted in dense submucosal fibrosis and altered tissue planes, thereby compromising adequate lifting. This observation underscores that poor lifting in GCP‑associated lesions may reflect the underlying pathology rather than necessarily indicating deep invasive carcinoma.

In the present case, the histopathological relationship between GCP and the coexisting early gastric cancer warrants careful consideration. The submucosal glands observed in Fig. 6 demonstrated benign cytological features—uniform nuclear morphology, preserved nuclear‑to‑cytoplasmic ratio, and absence of architectural atypia—and were therefore diagnosed as GCP rather than submucosal invasion by adenocarcinoma. The high‑grade intraepithelial neoplasia was confined to the surface and foveolar epithelium, without continuity with the submucosal cystic glands.

The diagnosis of early gastric cancer originating from GCP was based on the following evidence: (1) the close topographic association between the neoplastic mucosal epithelium and the underlying GCP, with the latter situated directly beneath the former; (2) the absence of conventional predisposing factors such as prior gastric surgery, making GCP the most plausible precursor lesion; (3) the well‑differentiated nature of the adenocarcinoma, which is consistent with the indolent clinical course observed over nine years; and (4) previous studies have documented the malignant potential of GCP, with some cases demonstrating transition from benign cystic glands to dysplastic epithelium. While it remains possible that HGIN arose incidentally in an area with pre‑existing GCP, the close spatial relationship and the absence of other mucosal pathologies strongly support a causal association.

The present case differs from previously reported GCP-associated EGC cases in several notable respects. First, the nine-year persistence at the HGIN stage exceeds the median duration of 44 months reported for EGC generally; however, as this observation is derived from a single case, it should be interpreted with caution and does not establish a generalizable pattern for GCP-associated lesions. Second, the diagnostic dilemma created by GCP-induced submucosal thickening on EUS—which led to treatment discontinuation and delayed definitive therapy—highlights a clinically important pitfall. While this phenomenon has been noted in previous reports, the present case underscores the practical challenges it poses in clinical decision-making. Third, the successful second ESD after nine years of observation in this single case suggests that some GCP-associated HGIN lesions may remain endoscopically resectable without progression over extended periods. However, this observation should not be interpreted as evidence supporting delayed intervention, and each case requires individualized management based on comprehensive endoscopic evaluation.

Several limitations of this report should be considered. As a single case observation, the findings cannot be generalized to the broader population of patients with GCP-associated lesions. Whether this patient‘s prolonged course reflects a broader tendency among GCP-associated lesions is unknown, and what contributed to it remains speculative. Additionally, the absence of systematic follow-up imaging during the nine-year interval limits our understanding of the lesion’s natural history during this period. The decision to delay definitive treatment was based on patient preference and clinical circumstances, and should not be construed as a recommended management approach. Future prospective studies with larger cohorts are needed to better define the natural history and optimal treatment strategies for GCP-associated early gastric cancer.

In conclusion, this case illustrates that GCP can be associated with well-differentiated early gastric cancer that may exhibit an indolent course over a prolonged period in some instances. Clinicians should be aware that EUS-detected submucosal thickening in GCP-associated lesions may mimic invasive carcinoma, and ESD remains a valuable diagnostic and therapeutic modality for these lesions. Longer-term observational data or pooled case series will be needed to clarify the natural history of GCP-associated gastric malignancies and inform optimal management.

## Data Availability

No datasets were generated or analysed during the current study.
